# New coating containing chitosan and *Hyssopus officinalis* essential oil (emulsion and nanoemulsion) to protect shrimp (*Litopenaeus vannamei*) against chemical, microbial and sensory changes

**DOI:** 10.1016/j.fochx.2023.100801

**Published:** 2023-07-18

**Authors:** Abbas Mehraie, Saied Khanzadi, Mohammad Hashemi, Mohammad Azizzadeh

**Affiliations:** a Department of Food Hygiene and Aquaculture, Faculty of Veterinary Medicine, Ferdowsi University of Mashhad, Mashhad, Iran; b Medical Toxicology Research Center, Mashhad University of Medical Sciences, Mashhad, Iran; c Department of Nutrition, Faculty of Medicine, Mashhad University of Medical Science, Mashhad, Iran; d Department of Clinical Science, Faculty of Veterinary Medicine, Ferdowsi University of Mashhad, Mashhad, Iran

**Keywords:** Antimicrobial properties, Antioxidant activity, Edible coating, Nanochitosan, Seafood

## Abstract

In this study, the effect of chitosan coating containing emulsion and nanoemulsion of *Hyssopus officinalis* essential oil (EO) on the chemical, microbiological and sensory characteristics of shrimp (*Litopenaeus vannamei*) was investigated. The minimum value of TVB-N (Total volatile basic nitrogen), TBARS (Thiobarbituric acid reactive substances), PV (peroxide value), TMA-N (Trimethylamine-nitrogen) and FFA (Free fatty acids) after 12 days were shown in NE + HEO 1% (coating containing chitosan with nanoemulsion of EO) with 20.53 mg N/100 g, 0.5 µg/kg, 0.88 MAQ peroxide/kg, 1.3 mg/100 g and 12.16 mg 100% of oleic acid, respectively. Also, minimum value of pH after 12 days was related to the CE + HEO 1% (coating containing chitosan with emulsion of EO) with 7.60. The minimum value of psychrophilic and mesophilic microbial count after 12 days were shown in NE + HEO 1%, 4.40 ± 0.36 and 4.03 ± 0.06 cfu/g, respectively. The best score of sensory evaluation was observed in the NE-HEO 1% treatment. As a result, the edible coating containing chitosan-based nanoemulsion could be effective to the preservation of shrimp's microbiological, chemical, and sensory characteristics.

## Highlights

• Chitosan coating containing nanoemulsions of *Hyssopus Officinalis* EO were prepared.
• The properties of the treated shrimp during storage at 4C were assessed.
• The chemical, microbiological and sensory properties were assessed.
• Shrimp samples treated with nanochitosan with 1% essential oil nanoemulsions maintained desirable properties longer than control or other treatments.

## 1 Introduction

Seafood products are recognized as valuable food all over the world due to their high nutritional value and deliciousness (Abdollahzadeh et al., 2014, Wu et al., 2015, Zlatanos and Laskaridis, 2007). Shrimp (*Litopenaeus vannamei*) is abundantly found in the Iranian market and this aquatic animal has a high nutritional value due to the combination of protein (percentage), minerals, etc. (Khodanazary, 2019). Aquatic products, especially shrimps, are among the valuable marine foods that are sensitive to spoilage and quickly become rotten and unusable due to the growth and proliferation of bacteria. Coating, freezing and cooling are often used to prevent microbiological, biochemical and physical degradation of shrimp. Also, new techniques such as modified atmosphere packaging (Asdagh et al., 2021), acidic electrolyzed water (Dib et al., 2018), irradiation, and added herbal extracts (Khodanazary, 2019, Pabast et al., 2018) are used today. In addition, with the increasing demand for low-processed foods without chemical preservatives, packaging with coatings (polysaccharide, protein, etc.) containing bioactive compounds is used today to maintain the quality and freshness of food (Khodanazary, 2019). Bioactive edible coatings are natural biopolymers derived from proteins, polysaccharides, and lipids, which act as a barrier against external factors such as aroma, oxygen, and moisture, and increase the quality and shelf life of food (Homayonpour, Jalali, Shariatifar, & Amanlou, 2021). As the most abundant amino polysaccharides in nature, chitin and chitosan have characteristics such as high biocompatibility, low toxicity, biodegradability, and acceptable antimicrobial properties (Sharma et al., 2022). Chitosan as a polycationic compound can inhibit a wide range of spoilage and pathogenic microorganisms such as yeasts, molds and Gram-negative and Gram-positive bacteria (Homayounpour, Shariatifar, & Alizadeh-Sani, 2021). Chitosan antimicrobial activity depends on the type of microorganism, molecular weight (approximately 50,000–190,000 Da), pH value, and deacetylation degree of the surrounding matrix (Qiu, Zhang, Chitrakar, Adhikari, & Yang, 2022). Also, Chitosan has intrinsic antioxidant properties, which are affected by its molecular weight and concentration (Donsì et al., 2011, Homayonpour et al., 2021, Keykhosravy et al., 2020). The genus Hyssopus includes aromatic, perennial plants found in parts of Europe, the Middle East, and around the Caspian Sea. The essential oil of these plants contains volatile compounds, mainly pinocamphen and pinene, and a glucoside called hysopine (Fathiazad & Hamedeyazdan, 2011). The Hyssop (*Hyssopus officinalis*) has a sharp and biting taste with a delightful smell that resembles the smell of mint. Hyssop essential oil has antibacterial and antioxidant properties due to its phenolic compounds (Fraternale et al., 2004, Kizil et al., 2010).

Combination of antioxidants into materials of packaging has become common since oxidation is a chief problem affecting the quality of food (Haghju et al., 2016, Homayounpour et al., 2020). Presently, the most regularly used antioxidants in active packaging are BHT (butylated hydroxytoluene) and BHA (butylated hydroxyanisole) (Siripatrawan and Harte, 2010, Taheri et al., 2018). Although these mentioned synthetic antioxidants can efficiently be used in active food packaging since of efficiency, low cost and high stability, there are significant worries associated to their aspects of toxicological (Siripatrawan and Harte, 2010, Taheri et al., 2018, Zhaveh et al., 2015). Additionally, the application of synthetic antioxidants is under severe regulation owing to the potential human health risk. Thus, widespread investigation has been conducted to employ some natural antioxidants like plant essential oils and chitosan as alternatives to synthetic antioxidants (Haghju et al., 2016, Homayounpour et al., 2020, Siripatrawan and Harte, 2010, Taheri et al., 2018, Zhaveh et al., 2015).

When plant essential oils are added to food, the activity of these compounds decreases due to their low solubility in water and their hydrophobic binding to nutrients such as fats and proteins (Aminzare et al., 2018, Hashemi et al., 2016, Hashemi et al., 2017, Javid et al., 2014, Raeisi et al., 2016). Various methods have been used to prevent this reduction, such as encapsulation and nanoemulsions (Donsì et al., 2011, Keykhosravy et al., 2020). The use of these methods causes the proper distribution of essential oils in food (Aminzare et al., 2017, Donsì and Ferrari, 2016).

However, some researchers used the EO mixture in coating solutions before sonication to make nanoemulsions (Donsì et al., 2011, Homayonpour et al., 2021, Keykhosravy et al., 2020). Reduced particle sizes of biopolymers (e.g., chitosan) should enhance their biological effectiveness and lead to better results in this regard (Donsì et al., 2011, Homayonpour et al., 2021, Keykhosravy et al., 2020). So far, studies have been conducted on the effect of different EO on the storage of various foods, but according to our knowledge, a proper study on HEO on shrimp storage has not been conducted. On the other hand, due to the high consumption of shrimp in the diet and its highly perishable nature (Khodanazary, 2019, Imran et al., 2013), the need for an extensive study to use different methods of preservation seems very necessary. In light of this, the present study aims to:

1. Analyzing HEO by gas chromatography-mass spectrometry and identifying its components.
2. To prepare HEO emulsions/nanoemulsions based on EO and to evaluate their characteristics.
3. To prepare and compare chitosan-loaded nanoemulsion/emulsion of HEO and investigate their effects on the chemical (pH, total volatile essential nitrogen, thiobarbituric acid reactive, peroxide value, trimethylamine nitrogen, and free fatty acid), microbiological (total psychrophilic and mesophilic) and sensory properties (odor, texture, color and overall) of shrimp stored at refrigeration (4 °C).

## 2 Materials and methods

### 2.1 Materials

From Merck Co. (Darmstadt, Germany), we purchased Folin-Ciocalteu reagent, sodium carbonate anhydrous, acetic acid, and PCA media (plate count agar). Sigma-Aldrich provided glycerol, Tween 80, methanol, NaOH, cholesterol 95%, dichloromethane, and other reagents and solvents of analytical grade or higher available purity. Chitosan with a distillation rate of 85% and a molecular weight of 1.6 × 10^5^ was purchased from SIGMA (medium molecular weight, Sigma-Aldrich Chemical Co.).

### 2.2 Preparation and analysis of hyssop plant essential oil compounds

Hyssop plant was collected from a local market in Tehran, Iran and sent to the Herbarium of the Biology Department of the Research Institute of Plants and Medicinal Raw Materials of Shahid Beheshti University of medical sciences (SBMU) for botanical approval (Herbarium no. HP-2567). The essential oil extraction process was performed using the Clevenger apparatus by distillation method. The total amount of hyssop plant used was about 5 kg. At each stage, 160 g of dried plant powder was poured into the Clevenger apparatus and about 480 mL of distilled water (with room temperature) was added to it. After 6 h, essential oil extraction is complete. Sodium anhydride sulfate was used to dehydrate the essential oil. To increase the contact surface of the plant with distilled water, the sample was ground with laboratory grinder (KM-100, MRC Co., UK) until it is completely crushed and uniform. The essential oil were kept in a dark glass bottle in the refrigerator (4 °C) until further experiments (Homayounpour et al., 2021).

Essential oil components were identified using a gas chromatography - mass spectrometer (the model of GC was Agilent 6890 (Palo Alto, CA, USA) with mass detector model Agilent 5973). The device was equipped with a DB-5 column (length 30 m, inner diameter 0.25 mm, inner layer thickness 0.25 µm), helium carrier gas with a flow of 1.1 mL/min, column temperature program was from 60 to 250 °C with the speed of 5 °C/min. The isolated compounds were identified following the mass spectrometer library (Saturn version 4; Agilent Technology Inc., Santa Clara, USA), the decomposition patterns presented in the other studies, and the calculation of the Quats index (Kizil et al., 2010, Soleymani and Pirzad, 2015). The relative amounts of components were determined according to the percentage of surface area under the peak (Fraternale et al., 2004, Homayounpour et al., 2021).

### 2.3 Preparation of solution/emulsion/nanoemulsion of chitosan coating containing HEO

For the preparation of chitosan (2% w/v) in acetic acid, chitosan was mixed in an aqueous solution of 1% acetic acid and stirred for 10 min. A NaOH solution of 1 N was added to the solution chitosan (to raise the pH of the prepared solution) and it was completely dissolved. Finally, the pH of the prepared solution was increased to 5.9 and then 2 mL of Tween 80 was added to one liter of solution (Keykhosravy et al., 2020).

To prepare the emulsion of chitosan coating containing HEO, 0.75 g of glycerol was added to the mixture as a plasticizer for each gram of chitosan and stirred for 10 min (Keykhosravy et al., 2020). An oily phase prepared by weighing 0.5 and 1 g of essential oil (hyssop) and mixing it with 0.2 g of tween 80 as a surfactant was mixed evenly. As a final step, the oily phase was added to an aqueous phase of chitosan (2 g) and blended. The mixture was mixed at room temperature until a homogeneous dispersion using a hot plate stereo magnet (IKA, C-MAG HS 10, Germany). At the end, the mixing of the emulsion was done at 10000 rpm in an Ultra-Turrax digital mixer for 2 min (IKA, Staufen, Germany) (Keykhosravy et al., 2020).

With high-intensity sonication at amplitudes of 50%, the emulsion was converted with a pulse of 45 s working and 15 s resting for six minutes (Sonopuls, Bandelin, Berlin, Germany). A water bath with ice is placed around the beaker containing the nanoemulsion so that the heat caused by the device's activity does not raise the temperature of the sample. The final concentration of the chitosan nanoemulsion coating containing hyssop includes acetic acid (0.01 mL/mL), chitosan (20 mg/mL), Tween 80 (2 mg/mL), glycerol (15 mg/mL) and EO (5 to 10 mg/mL) (Keykhosravy et al., 2020).

### 2.4 Preparation of shrimp samples and treatments

The shrimp were obtained from the shrimp breeding pond of Gomishan, Golestan Province, Iran, and transferred by cold chain to the food hygiene laboratory of Ferdowsi University, Mashhad, Iran. The shrimp samples had an average weight of 16 g and an approximate length of 150 mm. The shrimps were washed with distilled water (at room temperature and produced with a water distiller, model DragLab DS 4000, Germany) to remove foreign matter (soil, etc.), and 10 g samples were prepared under sterile conditions (by a sterile knife and gloves) and randomly divided into 10 groups; then the samples were immersed in different treatments for 1 min (as shown in Table 1). Then the samples were removed from the solution and placed on sterile mesh plates (100 µ, made of plastic) to drip the coating solution. After drying the coating in the laboratory, the samples were packaged in a plastic packet (Autoclavable Bags, made of polypropylene with 2 mm thickness) and kept for 12 days at 4 °C, and chemical, microbial and sensory analyzes were performed at intervals of 0, 2, 4, 8, and 12 days.

**Table 1:** Treatments list in the present study.

	Treatment	Description
1	CON	without any coating
2	SMS	Sodium Metabisulfite coating
3	CH	Immersion in chitosan 2% solution
4	NCH	Immersion in Sonicated chitosan solution
5	HEO 0.5%	*Hyssopus officinalis* essential oil (HEO) 0.5 %(W/V)
6	HEO 1%	*Hyssopus officinalis* essential oil (HEO) 1 % (W/V)
7	CE + HEO 0.5%	Chitosan emulsion coating containing 0.5 % (W/V) HEO
8	CE + HEO 1%	Chitosan emulsion coating containing 1 % (W/V) HEO
9	NE + HEO 0.5%	Chitosan nanoemulsion coating containing 0.5 % (W/V) HEO
10	NE + HEO 1%	Chitosan nanoemulsion coating containing 1 % (W/V) HEO

### 2.5 Measurement of the droplet size and polydispersity index (PDI)

Backscattered light was used to determine the PDI and droplet size. The measurement was performed utilizing a DLS system (dynamic light scattering) equipped with a backscatter detector and a laser diffractometer with a wavelength of 633 nm (25 °C) (Nano ZS, Malvern Instruments Ltd., the UK). In polystyrene cuvettes, samples were prepared (1:100) with bi-distilled water to prevent multiple scattering effects (Keykhosravy et al., 2020).

### 2.6 Microbiological analysis

Ten grams of shrimp sample was aseptically moved to a stomacher bag (Seward Stomacher circulator bag, Model No. 400, the UK), and 90 mL of normal saline (sterile, 0.85 %) was added. Then, the prepared sample was homogenized by a lab stomacher blender (Seward Stomacher 400 Circulator, UK) at 230 rpm for sixty seconds. With normal saline (in a ratio of 1:10 with NaCl (0.85 %) and distilled water), tenfold serial dilution was prepared and applied to analyze total psychrophilic and mesophilic counts. For this purpose, 0.5 mL of homogenates was spread evenly on the dry PCA media surface. The total mesophilic count was determined after incubating the plates for 48 h at 37 °C, and the total psychrophilic count was determined after incubating for 12 days at 7 °C (Mohan, Ravishankar, Lalitha, & Gopal, 2012).

### 2.7 Chemical quality evaluation

#### 2.7.1 The peroxide value (PV)

According to the Homayonpour et al. study, PV value was measured (Homayonpour et al., 2021). Five grams of samples are placed in a 250 flask and then add 10 mL of CHCl_3_-CH_3_COOH mixture (2:3) and shake until the fat enters the solution. Then 1 mL of saturated potassium solution was added. The lid was closed and put it in the dark place for 5 min. 20 mL of distilled water was added and then shaken. The released iodine was titrated with 0.01 mL Na_2_S_2_O_3_ solution until it turns bright yellow. One milliliter of 1.5% starch solution was added as indicator and was titrated until colorless.

#### 2.7.2 Value of pH

This method was carried out by homogenizing approximately 10 g of sample with twice the amount of distilled water (g/ml). A pH meter was used to determine the pH value (model HI 2002, HANNA Company, Germany). Experiments were performed at least three times (Homayonpour et al., 2021).

#### 2.7.3 TBARS assay

In this study, the TBARS (thiobarbituric acid reactive substances) assay was used according to the study of Pabast et al. (2018). In this research, shrimp (10 g) were homogenized at 4000 rpm for 2 min with 30 mL perchloric acid (4 %) and BHT (butylated hydroxytoluene) solution (1 mL) dissolved in ethanol. A Whatman filter (No. 4) was used to filter the mixture before use. In a stoppered test tube, 5 mL of the subsequent solution was mixed with 5 mL of TBA (0.02 M). As part of a water bath, the combination was kept at 95 °C for 60 min before being cooled in freezing water for 5 min. At the end of this method, sample treatments and control samples were read at a wavelength of 532 nm with a spectrophotometer (Ultrospec 2000, the UK) using a mixture of 5 mL TBA (0.02 M) and 5 mL perchloric acid (4%) as a sample blank.

#### 2.7.4 TMA-N assay

Determination of TMA-N was done on trichloroacetic acid (TCA) extracts of shrimp using the modified picric acid methods described by Mohan et al.'s study (Mohan et al., 2012). A Hewlett-Packard diode array spectrophotometer (model 8452A, Hewlett Packard Co.) was used to measure the absorbance of the organic phase combined with picric acid reagent at 410 nm. The content of TMA (µg) was estimated by multiplying the absorbance reading from a standard line made with standard TMA by a factor of 73.36. Using the equation below, we calculated the final TMA content in milligrams of TMA-N per 100 g of shrimp:

$$TMA\text{-}N=\frac{T[V1\;+\;(0.01MW)]}{V2W\;}\times 10$$

W = weight (g) of shrimp used in 1:2 extraction.
V_2_ = volume (mL) of extract added to test tube
V_1_ = volume (mL) of TCA added for 1:2 extraction
M = moisture of shrimp sample (in percent)
T = equivalent TMA in µg determined from a standard curve

#### 2.7.5 TVB-N (total volatile basic nitrogen) assay

Using the micro-diffusion method, total volatile basic nitrogen (TVB-N) of shrimp samples was determined in this study. The shrimp minced samples (10 ± 0.1 g) were homogenized in 100 mL of perchloric acid (6 %) for two minutes (in a suitable container) and then filtrated. Then it was alkalized with sodium hydroxide solution (20 %) and finally steam distillation of the extract is done. By an acid receiver, the volatile base components were absorbed and determined by titration. All analyses were carried out in triplicate. It was stated in mg N/100 g of shrimp samples for the results (Tometri, Ahmady, Ariaii, & Soltani, 2020).

#### 2.7.6 FFA assay

The value of free fatty acid (FFA) was determined by study of Reesha et al. to assess the hydrolytic rancidity (Reesha, Panda, Bindu, & Varghese, 2015). For this experiment, 75 mL of hot neutralized ethanol (95 %) and 2 mL solution of phenolphthalein indicator (one percent) were added to a 7.05 ± 0.05 g well-mixed shrimp sample. By adding a sodium hydroxide (0.25 N) solution to the ethanol, it was neutralized until a faint permanent pink color developed. After that, the shrimp samples were titrated against sodium hydroxide (0.25 N) until the first persistent pink color seemed, which was the same intensity as the neutralized ethanol before adding the sample. During the titration, the persistent pink color lasted at least 30 s. Equations were used to calculate the Free Fatty Acids content (percent FFA):

$$\mathrm{FFA}\;\%=\frac{mL\;of\;alkali\times N\times 28.2}{W}$$

Where: W is weight of oil (g), and N is normality of NaOH solution.

#### 2.7.7 Fatty acid profile analysis

A gas chromatography technique was used to determine the fatty acid profile of shrimp. The GC equipment (Agilent 7890 GC with FID & 7693 Autosampler) employed a flame ionization detector (FID) coupled to a cyanopropyl polyphenylene-siloxane column (BPX70, 30 m, 0.250 mm inner diameter, 0.25 µm film thickness and supplier Trajan Scientific and Medical, China) to determine the fatty acid profile of shrimp.

As a carrier gas, ultra-high-purity nitrogen (99.9% purity) was used at a flow rate of 1 mL/min.

As the first temperature, 140 °C, was planned as the column temperature for 5 min, followed by 240 °C at a rate of 4 °C/min. Temperatures of the injector and detector were set to 260 °C. For each analysis, a specific amount (1 µL) of the sample was injected. Under the same conditions, we determined the retention time of each F.A. and compared it to a mixture of fatty acid methyl esters obtained from an external commercial standard (F.A.M.E. Mix, C4-C24, catalog No. 189191AMP, Merck). We quantified the fatty acid contents and presented the results as a concentration ratio per total fatty acid in the samples.

### 2.8 Sensory evaluation

The consumer panel members evaluated the treated shrimp and the control samples for odor, color and texture. In this study, the method of Meilgaard et al. and Pabast et al. (with slight changes) was used on days 0, 2, 4, 8, 12 and with the 9-point hedonic scale (Meilgaard et al., 1991, Pabast et al., 2018). According to this method, the shrimp is considered acceptable if the sensory score is higher than 5.0. For this purpose, as judges, we selected 27 women and 25 men between the age of 27 to 45 years (Keykhosravy et al., 2021, Mohan et al., 2012). All panelists had a history in assessment of seafood before at University of Tehran, linked in other study exhibited by Noori, Zeynali, and Almasi (2018). The separate evaluation room was as follows: the temperature was 24 ± 1 °C (during the test, the room temperature was constant), the white and red fluorescence light provided the white light amount of 80 foot-candles of the table surface of the room. The light intensity was constant and uniform. Purified air under positive pressure was provided to create the air flow from the evaluation room to the surrounding environment. The test room was free of foreign odors. Separate rooms for evaluation were provided with separate 45 cm tables with long walls. Also, the walls and roof were light and matte and there were no unnecessary decorations. Finally, the number of samples for each evaluator was 10, and each test was repeated 3 times (Homayounpour et al., 2020, Kontominas et al., 2021, Pabast et al., 2018, Shahidi and Hossain, 2022).

### 2.9 Statistical analysis

All experiments were carried out in triplicate and were expressed as means ± SD (standard deviations). The chemical and microbial outcomes were compared between groups (treatments) by repeated measure ANOVA (repeated measure analysis of variance), followed by Duncan's post hoc test. Comparison of sensory indexes between groups evaluated using Friedman test. Pairwise comparisons were performed by Wilcoxon signed-rank test with Bonferroni correction. For pairwise comparisons P < 0.001 (Bonferroni-corrected P-value threshold) considered as significant. As statistically significant, a *p*-value < 0.05 was evaluated. Statistical analysis was performed by SPSS Ver.24 (Chicago, IL).

## 3 Results and discussion

### 3.1 Identification of compounds of *Hyssopus officinalis* essential oils (HEO)

The total percentage of 33 identified components of HEO was 99.75% (Table 2). The main components were Isopinocamphone, Pinocamphone, and β-Pinene, respectively. This research had similarities to the Fatemeh Fathiazad et al. study (Fathiazad & Hamedeyazdan, 2011). Also, Moro et al. assessed the compounds of HEO and reported that four compounds were the main compound in EO and had a maximum range (inclusive α-pinene, isopinocamphone, β-bourbonene, and β-caryophyllene) that α-pinene and isopinocamphone (as main compounds) were similar to this study (Moro, Zalacain, Hurtado de Mendoza, & Carmona, 2011).

**Table 2:** Chemical composition of *Hyssopus officinalis* essential oil by GC/MS.

	Compounds	Concentration (Peak area %)	RI (Retention Index)
1	α-Thujene	0.13	926
2	α-Pinene	0.59	934
3	Sabinene	0.98	975
4	β-Pinene	10.12	981
5	β-Myrcene	1.21	992
6	β-phellandrene	5.65	1032
7	E-β-Ocimene	0.14	1048
8	γ-Terpinene	0.15	1060
9	Linalool	1.14	1106
10	Pinocarveol<trans->	0.16	1147
11	Not identification	3.52	1161
12	Pinocamphone	11.81	1167
13	Isopinocamphone	35.45	1187
14	α-Terpinenol	0.27	1198
15	Myrtenol	0.88	1203
16	β-Bourbonone	1.73	1381
17	α-Gurjunene	0.50	1404
18	E-Caryophyllene	3.25	1416
19	α-Humulene	0.61	1450
20	Caryophyllene<9-*epi*-(E)->	1.94	1458
21	Germacrene D	3.68	1480
22	Bicyclogermacrene	4.04	1496
23	γ-Cadinene	0.17	1512
24	δ-Cadinene	0.24	1521
25	Elemol	5.11	1550
26	Spathulenol	1	1579
27	Caryophyllene oxide	0.6	1582
28	Globulol	0.29	1585
29	Ledol	0.31	1603
30	γ-Eudesmol	1.47	1632
31	δ-Cadinol	0.53	1640
32	β-Eudesmol	0.80	1651
33	α-Eudesmol	1.28	1654
	Total	99.75	

### 3.2 Characterization of treatments

Table 3 shows the characterization results of CH, NCH, HEO 0.5 %, HEO 1 %, CE + HEO 0.5 %, CE + HEO 1%, NE + HEO 0.5 % and NE + HEO 1 % in the experimental groups. The treatments were prepared with particle sizes ranging from 342.33 ± 2.51 to 4400.10 ± 187.50 nm. The ultrathrux rotation speeds associated directly with the diameter of the droplet and stability of the emulsion. Similar to the Mendes et al. research (Mendes et al., 2020), increasing the speed to 12,000 rpm reduced droplet diameter. At 0.5% surfactant concentration, the average diameter of emulsion droplets decreased. The size of the chitosan solution was 4400.10 ± 187.50 nm. Afterward, chitosan solution was sonicated with tween 80, and droplets size was reduced to 800.21 ± 67.73 nm, which showed sonication had reduced the particle size, which was similar to the previous research (Mendes et al., 2020). A chitosan-loaded nanoemulsion containing 0.5 % HEO (342.33 ± 2.51 nm) had a smaller means droplet diameter than the other treatments. (As a surfactant) Tween 80 was used because it has a high hydrophilic-lipophilic balance (HLB-15) that makes it suitable for emulsions of oil-in-water (Donsì & Ferrari, 2016). The polydispersity index (PDI) measures the heterogeneity of particles or molecule sizes, and this index ranges from 0 to 1. The minimum PDI index was related to CH treatment (0.26 ± 0.02), and the maximum index was related to NCH (0.46 ± 0.06).

**Table 3:** Characterization of treatments (particle size, PDI and Z-potential).

**Treatments**	**Particle size** **(nm)**	**PDI (polydispersity index)**	**Z-Potential**
**CH**	4400.10 ^a^ ± 187.50	0.26 ^a^ ± 0.02	56.92 ^a^ ± 2.32
**NCH**	800.21 ^b^ ± 67.73	0.46 ^b^ ± 0.06	47.22 ^b^ ± 1.68
**HEO 0.5%**	1123.87 ^c^ ± 17.43	0.38 ^c^ ± 0.02	49.34 ^c^ ± 3.1
**HEO 1%**	1214.76 ^d^ ± 24.33	0.33 ^d^ ± 0.03	47.23 ^d^ ± 1.22
**CE + HEO 0.5%**	1350.54 ^e^ ± 41.23	0.41 ^e^ ± 0.03	43.87 ^e^ ± 2.5
**CE + HEO 1%**	1420.08 ^f^ ± 13.25	0.36 ^f^ ± 0.04	41.45 ^f^ ± 1.66
**NE + HEO 0.5%**	342.33 ^g^ ± 2.51	0.39 ^g^ ± 0.01	52.00 ^g^ ± 2.64
**NE + HEO 1%**	455.13 ^h^ ± 4.46	0.31 ^h^ ± 0.03	40.63 ^h^ ± 1.45

The same uppercase letter within a column are not statistically different (p < 0.05).

The zeta-potential ranged from 40.63 ± 1.45 to 56.92 ± 2.32. If the absolute value of the zeta potential is in the range of 30 to 60 mV, the nanoemulsion system is stable, with lower values indicating greater stability (Keykhosravy et al., 2020). This research used nanoemulsions combined with essential oil, which are used as coating solutions prior to the sonication process. The achieved results of the present research exhibited stable systems and were similar to the findings of Salvia- Trujillo et al. (Salvia-Trujillo, Rojas-Graü, Soliva-Fortuny, & Martín-Belloso, 2015) and other studies (Donsì and Ferrari, 2016, Keykhosravy et al., 2020, Mendes et al., 2020).

### 3.3 Chemical quality evaluation

#### 3.3.1 Value of pH

One indicator of the freshness assessment of shrimp and other seafood products is determining pH value. Changes in pH values of samples were demonstrated in Table 4. At the beginning of the test, the pH was almost equal. The trend of pH levels during the experiments was increasing for all treatments and control samples. The average pH during the study period in the treatment groups was lower than the control group. The best pH performance was related to CE + HEO 1% treatment, which was statistically significantly different compared to all treatments. Also, NE + HEO with both 0.5 and 1 % levels had better effects but lower effect than CE + HEO at 1%. Sodium metabisulfite had a better effect than the control group, which was not significantly different from other treatments. HEO 0.5 and 1 % had a good effect on pH value compared to the control group, although it had a lower effect than CE + HEO and NE + HEO. The highest pH value was assessed in the control sample (8.19 ± 0.01) that the increase in the pH in samples might be associated with the production of alkaline substances (such as ammonia, trimethylamine, indole, and histamine), autolysis process, endogenous enzymes (lipases and proteases) or microbial enzymes activity that leads to an increase in volatile bases during long-term storage (Homayounpour et al., 2021, Kizil et al., 2010, Noori et al., 2018, Shahbazi, 2017). It is also thought that the increase in pH during storage at cold temperatures is due to the production of amines by amino acid decarboxylation (Aşik and Candoğan, 2014, Homayounpour et al., 2021, Kizil et al., 2010, Noori et al., 2018, Shahbazi, 2017). Also, the relationship between the pH index and the number of psychrophilic and mesophilic microorganisms evaluated by the Pearson test confirms the significant relationship between the increase in the number of microorganisms and the increase in pH (Aşik and Candoğan, 2014, Homayounpour et al., 2021, Hussain et al., 2021, Kizil et al., 2010, Razavi et al., 2020). This study was similar to the Homayonpor et al. study. They reported that nano form of *Cuminum cyminum L*. EO had a better effect than the free form of EO during storing at 4 °C (Homayounpour et al., 2021). Also, Pabst et al. reported that nano encapsulated *Satureja* plant EO had a better effect on the pH of lamb meat than EO's free form during storing at 4 °C (Pabast et al., 2018).

**Table 4:** Changes in pH values with different treatment.

	Day 0	Day 2	Day 4	Day 8	Day 12
CON	7.42^aA^ ± 0.12	7.70 ^aB^ ± 0.17	7.70 ^aB^ ± 0.14	8.15 ^aC^ ± 0.23	8.19 ^aD^ ± 0.16
**CH**	7.40^bA^ ± 0.10	7.67 ^bB^ ± 0.12	7.67 ^bB^ ± 0.16	7.75 ^bC^ ± 0.12	7.89 ^bD^ ± 0.21
**NCH**	7.44^cA^ ± 0.14	7.58^cB^ ± 0.15	7.66 ^bC^ ± 0.12	7.74 ^bD^ ± 0.07	7.70 ^cB^ ± 0.12
**SMS**	7.43^cA^ ± 0.09	7.61 ^dB^ ± 0.08	7.60 ^cB^ ± 0.12	7.63 ^cC^ ± 0.15	7.78 ^dD^ ± 0.13
**HEO 0.5%**	7.40^bA^ ± 0.13	7.59 ^cB^ ± 0.13	7.58 ^dB^ ± 0.15	7.72 ^dC^ ± 0.14	7.70^cD^ ± 0.09
**HEO 1%**	7.35^dA^ ± 0.12	7.51 ^eB^ ± 0.21	7.63 ^fC^ ± 0.08	7.64^cD^ ± 0.13	7.62 ^eD^ ± 0.16
**CE + HEO 0.5%**	7.31^eA^ ± 0.08	7.39 ^fB^ ± 0.08	7.54 ^eC^ ± 0.14	7.68 ^eD^ ± 0.19	7.71^cD^ ± 0.15
**CE + HEO 1%**	7.36^dA^ ± 0.11	7.41 ^gB^ ± 0.12	7.48 ^hC^ ± 0.21	7.55 ^fD^ ± 0.12	7.60 ^fD^ ± 0.14
**NE + HEO 0.5%**	7.31^eA^ ± 0.08	7.43 ^hB^ ± 0.14	7.52 ^gC^ ± 0.12	7.61 ^gD^ ± 0.13	7.68 ^gD^ ± 0.17
**NE + HEO 1%**	7.32^eA^ ± 0.16	7.66 ^bB^ ± 0.22	7.43 ^hC^ ± 0.09	7.56 ^fD^ ± 0.08	7.64 ^hD^ ± 0.09

The same uppercase letter within a column or the same lower-case letter within a row are not statistically different (p < 0.05).

#### 3.3.2 TVB-N

**Fig. 1a:**
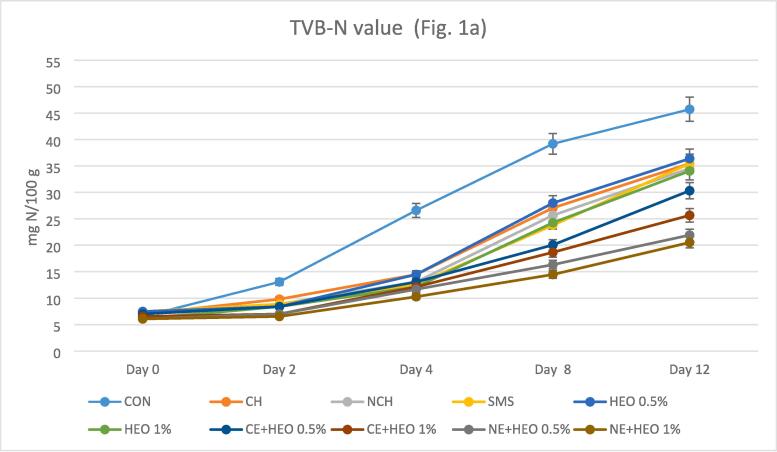

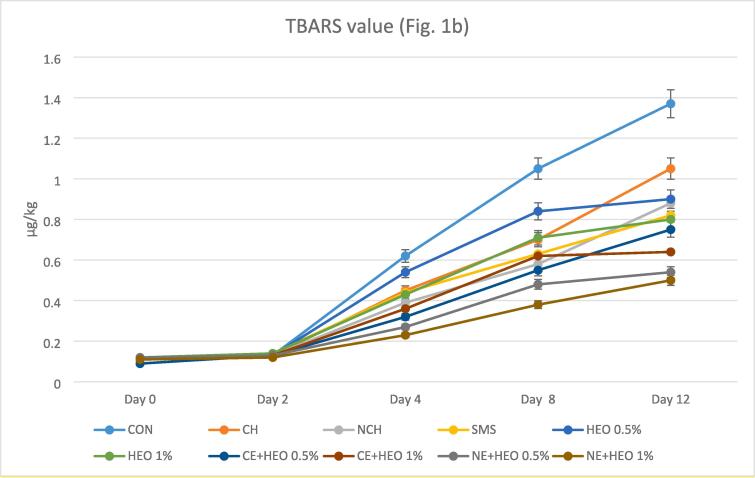

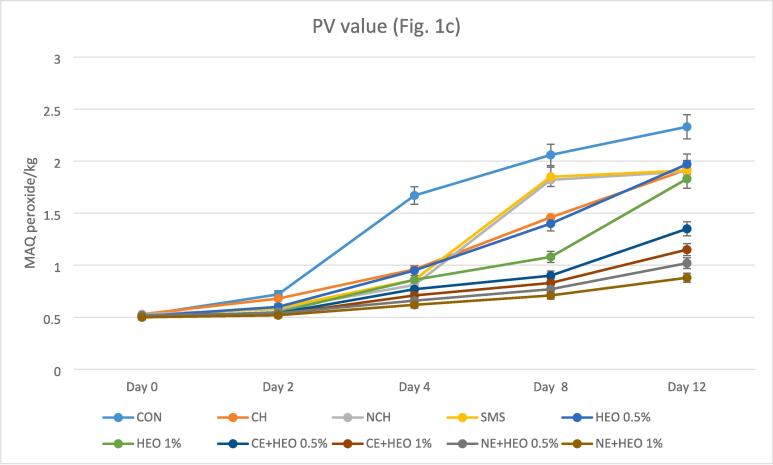

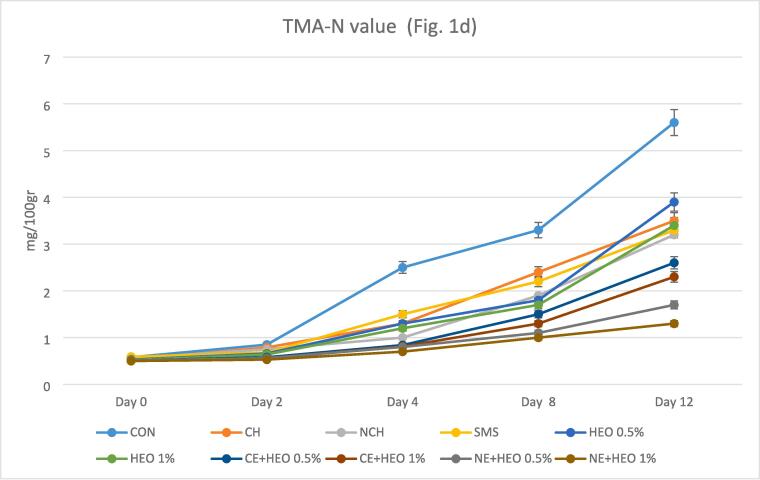

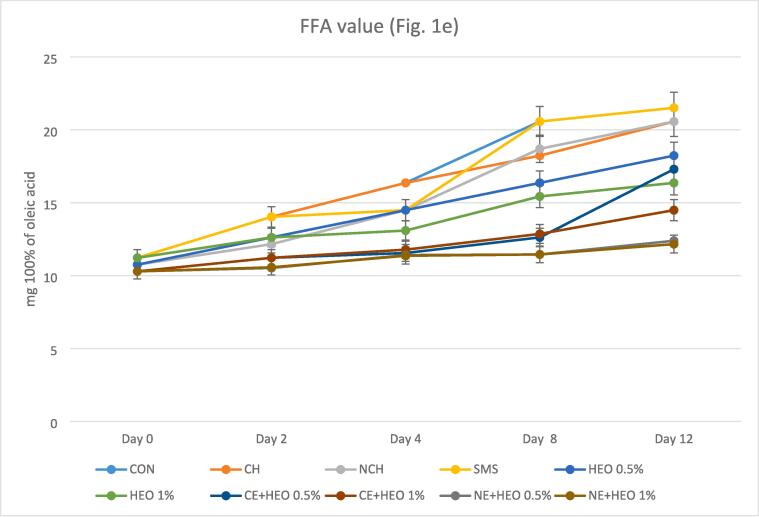
Changes in TVB-N (total volatile basic nitrogen); Fig. 1b. Changes in TBARS value (Thiobarbituric acid reactive substances); Fig. 1c. Changes in PV (peroxide value); Fig. 1d. Changes in TMA-N (Trimethylamine-nitrogen); Fig. 1e. Changes in FFA (Free fatty acids) values with different treatments. In all figures, the lower the test value on the last day, the better the treatment or the higher quality (so that in all figures, the control sample had the lowest quality). Treatments include: control (CON) without coating, Sodium Metabisulfite coating (SMS), coating containing chitosan (CH) and nanochitosan (NCH), coating containing essential oil (HEO, 0.5 and 1 %), coating containing chitosan with emulsion (CE + HEO, 0.5 and 1 %) and coating containing chitosan with nanoemulsion (NE + HEO, 0.5 and 1 %).

The TVB-N levels depend on the freshness and quality of the shrimp samples (Imran et al., 2013, Jinadasa, 2014). Fig. 1a represents the comparison of TVB-N during the 12 days. At the beginning of the test, the amount of TVB-N was almost equal, with minor differences. The mean of TVB-N changes in all treatments was lower than the control group. This indicates the good performance of all treatments compared to the control group during 12 days of storage. Sodium metabisulfite had a better performance than the control group, which was not significantly different from other treatments. HEO with both 0.5 and 1% levels had a good function on TVB-N compared to the control group, but it was less effective than CE + HEO and NE + HEO and compared to other treatments, it was not significantly different. The best performance on TVB-N belonged to the NE + HEO 0.5 and 1 %, which had good effectiveness compared to all treatments and had a statistically significant difference of 0.05%. Although the function of NE + HEO 1% was better, this difference was not significant with NE + HEO 0.5 %. TVB-N of CE + HEO treatment with both levels of 0.5 and 1% performed well, but NE + HEO ratio of 1% was better than others and it was not significantly different from other treatments. The highest amount of TVB-N was evaluated in the control samples (45.73 mg/100 g). With increasing storage time, the amount of TVB-N increased, which was due to the degradation of proteins and derivatives, the degradation of the enzyme in the shrimp and also producing several volatile bases like H_2_S, histamine, ammonia and foul-smelling trimethylamine (Aşik and Candoğan, 2014, Reesha et al., 2015). Several factors such as the activity of bacterial species, the activity of internal enzymes and the production of alkaline metabolites from the multiplying microorganisms or the excretion of proteins are involved in increasing the level of TVB-N (Homayounpour et al., 2020, Imran et al., 2013, Jinadasa, 2014). The use of HEO and chitosan in the form of emulsion and nanoemulsion can have a positive effect in preventing the increase of TVB-N due to preventing the activity of microorganisms (Donsì and Ferrari, 2016, Keykhosravy et al., 2020). Tometri et al., reported that nano form of *Laurus nobilis* leaf extract had a better effect than free form, which was similar to this research (Tometri et al., 2020). Hasani et al. reported nano-encapsulation form of EO had a better effect than lemon EO's free form in the TVB-N assay, which confirmed the Tometri study and our results (Hasani, Ojagh, Ghorbani, & Hasani, 2020).

#### 3.3.3 Peroxide value

Using the PV index, it is possible to measure the products resulting from the initial oxidation of fats and unsaturated oils (Homayounpour et al., 2020, Homayounpour et al., 2021, Pabast et al., 2018, Shariatifar et al., 2019, Taheri et al., 2018). Fig. 1c shows different levels of PV index in different treatments. At the beginning of the experiment, the PV value was almost equal, with minor differences. During the storage period, the PV levels increased significantly in the samples, which was caused by the formation of short-chain fatty acids owing to the microbial enzymes hydrolysis that is sensitive to oxidation and conducted to the peroxides formation (Aşik and Candoğan, 2014, Homayounpour et al., 2021, Kizil et al., 2010, Pabast et al., 2018). The PV in the treatments ranged from 0.50 to 2.33 meq peroxide/kg. The mean PV changes of all treatments were significantly lower than the control group. This indicates that all treatments performed well compared to the control group during 12 days (except CE + HEO 0.5%). Sodium metabisulfite had better function only than the control group but compared to HEO 1%, CE + HEO 0.5 and 1%, and NE + HEO 0.5 and 1%, had lower effectiveness and was not significantly different from other treatments. HEO 1% had good performance on PV compared to the control group, sodium metabisulfite, and NCH, although it had lower effectiveness than CE + HEO and NE + HEO and was not significantly different from other treatments. The best performance on PV belonged to the NE + HEO 0.5 and 1%, which had an excellent function compared to all treatments and had a significant difference at p < 0.05. Although the effectiveness of NE + HEO was better with 1% of essential oil, this difference was not significant with 0.5% of essential oil. CE + HEO had good performance on PV with both 0.5 and 1% levels but were lower than NE + HEO's 1% and were not significantly different from other treatments. All of the samples in nano-form showed a lower amount of PV, and treatment with *Hyssopus officinalis* had a better effect in all samples, attributed to the presence of effective phenolic compounds in *Hyssopus officinalis* essential oil. The increase of PV compounds may be related to the existence of fatty acids in the shrimp muscles that have been oxidized in storage, leading to the formation of peroxide or hydroperoxide. In addition, the removal of hydrogen from the double bond of fatty acids generates free fatty acid radicals that can react with O_2_ to produce hydroperoxide of fatty acids (Homayounpour et al., 2021, Kizil et al., 2010, Pabast et al., 2018).

#### 3.3.4 TBARS value

The TBARS value is one of the critical indicators restrictive the shelf life of shrimp and other seafood, which determines the secondary oxidation products associated with oxidative rancidity (Homayounpour et al., 2020, Kontominas et al., 2021, Pabast et al., 2018, Shahidi and Hossain, 2022). Fig. 1b shows the amount of TBARS in different treatments. At the beginning of the experiment, the TBARS values were almost the same. Gradually the differences in the TBARS values of different treatments were observed. The primary TBARS values of treatments were the lowest (0.12 mg MDA/kg). This shows the good performance of all coatings compared to the control group during 12 days of storage. The mean TBARS changes in all treatments were lower than the control group. Sodium metabisulfite had better effectiveness only than the control group, with no significant difference compared to other treatments. HEO 0.5 and 1% had a good function on TBARS compared to the control group, although HEO had a lower performance than NE + HEO and was not significantly different from other treatments. The best performance on TBARS belonged to the NE + HEO 0.5 and 1%, which had a perfect function compared to all treatments and had a statistically significant difference at the level of p < 0.05%, although the performance of NE + HEO was better with 1% of essential oil, this difference was not significant with 0.5% essential oil. CE + HEO 0.5 and 1% had a good effect on TBARS, although it had a lower function than NE + HEO 1% and was not significantly different from other treatments. All of the samples in nano-form showed a lower amount of TBARS, and treatment with *Hyssopus officinalis* had a better effect in all samples. In shrimp, peroxidation of lipids can be started by photosensitized oxidation and autoxidation or by using reaction enzymatic, like those relating to peroxidase, lipoxygenase, and enzymes of microbial (Aşik and Candoğan, 2014, Homayounpour et al., 2021, Kizil et al., 2010, Pabast et al., 2018). The reduced lipid oxidation in chitosan-coated pink salmon (Sathivel, Liu, Huang, & Prinyawiwatkul, 2007), herring and Atlantic cod (Jeon, Kamil, & Shahidi, 2002), and double filleted Indian oil sardine (*Sardinella longiceps*) (Mohan et al., 2012) have also reported that similar to this study.

#### 3.3.5 Trimethyleamine (TMA-N)

In improper shrimp storage conditions, trimethylamine oxide can be decomposed by existing bacteria or enzymes under the influence of acidic conditions, resulting in the formation of TMA-N (Abdeldaiem et al., 2017, Erkan et al., 2011, Malinowska-Pańczyk and Kołodziejska, 2016). At the beginning of the experiment, the amount of TMA-N was almost equal, with minor differences. It has been suggested that the maximum acceptable level of TMA-N in shrimp and other seafood is approximately 5 mg/100 g. In the current study, the TMA-N values of the samples ranged from around 0.50 mg/100 g up to 5.60 mg/100 g (Fig. 1d). Overall, TMA-N of the control sample exhibited the highest amount during the period of the experiment (P ≤ 0.05), so it reached 5.60 mg/100 g on the 12th day. On the contrary, NE-HEO 1% of treatments possessed the lowest TMA-N (1.30 mg/100 g) on the 12th day. All of the treatments in nano-form displayed a lower amount of TMA-N, and the treatment with *Hyssopus officinalis* had a better effect in all samples. The mean change in TMA-N for all treatments was lower than the change in the control group. Compared to the control group, all treatments performed well during storage for 12 days. Sodium metabisulfite had a better performance than the control group, which was not significantly different from other treatments. HEO with both 0.5 and 1% levels had a good performance on TMA-N compared to the control group; but it was less effective than CE + HEO and NE + HEO and compared to other treatments, it was not significantly different. The best performance on TMA belonged to the NE + HEO 0.5 and 1 %, which had a good performance compared to all treatments and had a statistically significant difference of 0.05%. Although the function of NE + HEO 1% was better, this difference was not significant with NE + HEO 0.5 %. CE + HEO 0.5 and 1% performed well on TMA-N but were lower than NE + HEO 1% and were not significantly different from other treatments. Control samples had the highest TMA value (5.6 mg/100 g) which the increase in compounds of TMA-N may be associated with the degradation of non-protein and proteins via spoilage of bacterial shrimp (Aşik and Candoğan, 2014, Kizil et al., 2010, Mohan et al., 2012, Reesha et al., 2015). According to Jeon et al. chitosan edible film used for quality preservation significantly reduced TMA-N content for cod and herring samples (Jeon et al., 2002).

#### 3.3.6 FFA value

The initial content of FFA of treatments was 10.29–11.22 mg % oleic acid, and it raised gradually to 21.51–12.16 mg % oleic acid on the last day of storage (Fig. 1e). At the beginning of the test, the amount of FFA was almost equal, with minor differences. The FFA content of samples increased during the storage period. At the end of the period (12 days), the highest FFA value was observed in the control sample (21.51 mg % oleic acid), and the lowest FFA value was observed in the NE-HEO 1% sample (12.16 mg % oleic acid). There is a significant difference between the mean changes of FFA in HEO 1%, CE + HEO 0.5%, CE + HEO 1% and NE + HEO 0.5%, NE + HEO 1% compared to the control group. This indicates the good performance of these treatments compared to the control group during 12 days of storage. Sodium metabisulfite had lower effectiveness than CE + HEO 1%, NE + HEO 0.5% and NE + HEO 1%, which had no significant difference compared to other treatments. HEO 0.5% and HEO 1% had good effect on FFA compared to the control group. HEO 0.5% had lower performance than CE + HEO 1% and NE + HEO 0.5%, which was not significantly different from other treatments. HEO 1% had better function than the control group and NCH and had lower performance than NE + HEO 0.5%, which was not significantly different from other treatments. The best performance on FFA belonged to the NE + HEO 0.5% and 1 %, which were very good compared to all treatments and had a statistically significant difference at 0.05%. CE + HEO 0.5 % and CE + HEO 1% had good performance on FFAs but was lower than NE + HEO 0.5%. All of the samples in nano-form (except NCH) showed a lower amount of FFA, and treatment with *Hyssopus officinalis* had a better effect in all samples that were owing to the hydrolysis and oxidation of lipids and was undesirable since the fatty acids may be changed to odorous volatiles (Mohan et al., 2012, Reesha et al., 2015). A change in FFA value similar to our study was reported by Reesha et al. in chilled fish (with chitosan film) during storage time (Reesha et al., 2015).

#### 3.3.7 Fatty acid profile

Due to its high unsaturated fatty acid content, shrimp is very sensitive to the oxidation process. Most of this fat is located under the skin, which makes the unsaturated fatty acids vulnerable to oxidation if exposed to light and oxygen (Lalitha & Gopal, 2012; Reesha et al., 2015). Also, due to the activity of microorganisms, oxidation of fats and also the formation of hydroperoxides, the content of fatty acids changes (Mohan et al., 2012, Reesha et al., 2015). As shown in Table 5, the percentage of unsaturated fatty acids (UFAs) was higher than saturated fatty acids (SFAs) in the lipid profile analysis of shrimp. The percentage of SFA and MUFA increased during storage, while the PUFA content decreased. PUFA/SFA were therefore declined during storage. An earlier study (Keykhosravy et al., 2021) found similar patterns of changes. At the beginning of the storage period, the ω-6 and ω-3 PUFAs, which are the most preferred for nutritional purposes (D’Angelo et al., 2020, Kapoor et al., 2021, Silva et al., 2014), were at their maximum amount. On the 12th day of storage, both ω-3 and ω-6 contained the minimum amount of content, which was 8.7–0.8 and 3.7–0.4 on the first day of storage, respectively.

**Table 5:** Changes in Fatty acid profile with different treatment.

Day/Treatment	**SFA**		**MUFA**		**PUFA**		**ɷ3**		**ɷ6**	
	**DAY0**	**DAY12**	**DAY0**	**DAY12**	**DAY0**	**DAY12**	**DAY0**	**DAY12**	**DAY0**	**DAY12**
**CON**	69.0 ^aA^ ± 0.02	78.4 ^aB^ ± 00.42	21.6 ^aA^ ± 0.09	27.0 ^aB^ ± 0.11	13.1 ^aA^ ± 0.55	8.2 ^aB^ ± 0.016	0.8 ^aA^ ± 0.02	0.4 ^aB^ ± 0.06	8.7 ^aA^ ± 0.34	3.7 ^aB^ ± 0.04
**CH**	68.4 ^bA^ ± 0.53	73.7 ^bB^ ± 0.23	21.7 ^aA^ ± 0.44	26.3 ^bB^ ± 0.27	14.3 ^bA^ ± 0.02	9.6 ^bB^ ± 0.21	0.7 ^bA^ ± 0.02	0.6 ^bB^ ± 0.03	8.1 ^bA^ ± 0.06	4.7 ^bB^ ± 0.16
**SMS**	68.6 ^bA^ ± 0.84	72.6 ^cB^ ± 0.04	20.2 ^bA^ ± 0.41	22.6 ^cB^ ± 0.22	13.8 ^cA^ ± 0.22	11.5 ^cB^ ± 0.27	0.8 ^cA^ ± 0.02	0.6 ^bB^ ± 0.03	8.0 ^bA^ ± 0.12	5.0 ^cB^ ± 0.16
**NCH**	68.2 ^bA^ ± 0.66	74.1 ^dB^ ± 0.14	19.9 ^cA^ ± 0.62	25.0 ^dB^ ± 0.08	13.7 ^cA^ ± 0.41	10.1 ^dB^ ± 0.17	0.8 ^cA^ ± 0.03	0.6 ^bB^ ± 0.02	7.7 ^cA^ ± 0.35	5.1 ^cB^ ± 0.07
**HEO 0.5%**	68.6 ^bA^ ± 0.11	77.5 ^eB^ ± 0.47	20.8 ^aA^ ± 0.16	26.1 ^bB^ ± 0.17	14.4 ^bA^ ± 0.13	10.2 ^dB^ ± 0.17	0.8 ^cA^ ± 0.04	0.5 ^cB^ ± 0.10	7.7 ^cA^ ± 0.23	3.3 ^dB^ ± 0.019
**HEO 1%**	69.5 ^bA^ ± 0.54	74.2 ^dB^ ± 0.15	20.0 ^bA^ ± 0.52	23.5 ^eB^ ± 0.26	14.0 ^dA^ ± 0.34	12.3 ^eB^ ± 0.17	0.8 ^cA^ ± 0.03	0.4 ^dB^ ± 0.02	8.1 ^bA^ ± 0.06	5.7 ^eB^ ± 0.3
**CE + HEO 0.5%**	68.5 ^bA^ ± 0.25	72.8 ^cB^ ± 0.13	19.9 ^cA^ ± 0.43	24.2 ^fB^ ± 0.11	14.5 ^bA^ ± 0.12	10.9 ^fB^ ± 0.53	0.8 ^cA^ ± 0.02	0.6 ^eB^ ± 0.06	7.6 ^cA^ ± 0.16	5.7 ^eB^ ± 0.29
**CE + HEO 1%**	69.7 ^aA^ ± 0.21	72.1 ^cB^ ± 0.10	20.6 ^cA^ ± 0.26	23.3 ^eB^ ± 0.06	14.4 ^bA^ ± 0.31	11.3 ^gB^ ± 0.11	0.9 ^dA^ ± 0.01	0.5 ^fB^ ± 0.02	8.8 ^dA^ ± 0.27	6.8 ^fB^ ± 0.11
**NE + HEO 0.5%**	68.3 ^aA^ ± 0.9	69.8 ^dB^ ± 0.03	19.7 ^cA^ ± 0.45	21.2 ^fB^ ± 0.17	14.3 ^bA^ ± 0.07	12.1 ^hB^ ± 0.16	0.8 ^cA^ ± 0.02	0.7 ^gB^ ± 0.02	8.7 ^dA^ ± 0.08	7.6 ^gB^ ± 0.2
**NE + HEO 1%**	68.0 ^bA^ ± 0.52	69.1 ^dB^ ± 0.05	19.9 ^cA^ ± 0.41	21.1 ^fB^ ± 0.03	14.2 ^bA^ ± 0.12	12.4 ^hB^ ± 0.06	0.9 ^dA^ ± 0.04	0.8 ^hB^ ± 0.02	8.3 ^eA^ ± 0.04	7.5 ^gB^ ± 0.20

The same uppercase letter within a column or the same lower-case letter within a row are not statistically different (p < 0.05).

### 3.4 Microbiological analysis

Table 6 represents the changes in total mesophilic and psychrophilic during storage for treated and untreated (control) shrimp samples. The primary quality of the shrimp used in the research was promising, showing low primary counts (2.37–2.53 cfu/g for psychrophilic bacteria and 3.07–3.47 cfu/g for mesophilic bacteria) in treatments during the storage time; the counts exhibited an increasing trend. The rise was higher for control samples compared to the other treatments. A relative log phase shows the efficiency of chitosan, HEO, and NE-HEO in inhibiting the microorganism’s growth. All of the treatments in nano-form exhibited a lower amount of total psychrophilic and mesophilic counts, and treatment with HEO had a better effect in all samples. The control group had maximum growth of bacteria (8.33 ± 0.25 cfu/g for Psychrophilic and 7.47 ± 0.35 cfu/g for mesophilic at the end of experiments), and NE + HEO 1% had minimum growth of bacteria (4.40 ± 0.36 cfu/g for Psychrophilic and 4.03 ± 0.06 cfu/g for mesophilic at the end of experiments) that was due to the many reasons such as its function as a barrier against O_2_ transfer, lipopolysaccharide layer disruption of the bacteria (outer membrane), the interaction with groups of anionic on the cell surface and antibacterial effect of chitosan and HEO (due to the phenolic and other antibacterial compounds) (Mohan et al., 2012). A similar reduction log cycle was reported for chitosan treated in fish patties by López-Caballero, Gómez-Guillén, Pérez-Mateos, and Montero (2005), in cod and herring by Jeon et al. (2002), and in chilled fish by Reesha et al. (2015).

**Table 6:** Changes in total psychrophilic and mesophilic counts for shrimp samples with various treatment during the storage time.

**Treatment**	**Psychrophilic (cfu/g)**					**Mesophilic (cfu/g)**				
	**Day 0**	**Day 2**	**Day 4**	**Day 8**	**Day 12**	**Day 0**	**Day 2**	**Day 4**	**Day 8**	**Day 12**
**CON**	2.53 ^aA^ ± 0.21	4.73 ^aB^ ± 0.15	6.57 ^aC^ ± 0.31	7.50 ^aD^ ± 0.36	8.33 ^aE^ ± 0.25	3.27 ^aA^ ± 0.21	4.70 ^aB^ ± 0.26	5.80 ^aC^ ± 0.17	6.43 ^aD^ ± 0.12	7.47 ^aE^ ± 0.35
**CH**	2.53 ^aA^ ± 0.12	3.83 ^bB^ ± 0.06	5.27 ^bC^ ± 0.06	6.27 ^bD^ ± 0.25	6.93 ^bE^ ± 0.15	3.47 ^bA^ ± 0.12	3.93 ^bB^ ± 0.06	4.27 ^bC^ ± 0.21	5.33 ^bD^ ± 0.15	6.33 ^bE^ ± 0.15
**NCH**	2.43 ^bA^ ± 0.12	3.50 ^cB^ ± 0.10	3.80 ^cC^ ± 0.15	4.40^cD^ ± 0.36	6.73 ^cE^ ± 0.29	3.20 ^cA^ ± 0.26	3.93 ^bB^ ± 0.12	4.37 ^cC^ ± 0.23	5.20^cD^ ± 0.10	6.27^cD^ ± 0.06
**SMS**	2.43 ^bA^ ± 0.06	3.17 ^dB^ ± 0.12	4.43 ^dC^ ± 0.32	5.17 ^dD^ ± 0.29	6.03 ^dE^ ± 0.23	3.27 ^aA^ ± 0.31	3.60 ^cB^ ± 0.26	4.03 ^dC^ ± 0.15	5.07 ^dD^ ± 0.06	5.87 ^dE^ ± 0.06
**HEO 0.5%**	2.52 ^aA^ ± 0.20	3.77 ^eB^ ± 0.12	4.27 ^eC^ ± 0.06	5.23 ^eD^ ± 0.12	6.73 ^cE^ ± 0.21	3.37 ^dA^ ± 0.15	4.07 ^dB^ ± 0.12	4.53 ^ec^ ± 0.21	5.63 ^eD^ ± 0.15	6.57 ^cE^ ± 0.32
**HEO 1%**	2.37 ^cA^ ± 0.06	3.17 ^dB^ ± 0.06	4.33 ^fC^ ± 0.29	4.83 ^fD^ ± 0.21	6.30 ^eE^ ± 0.10	3.27 ^aA^ ± 0.12	3.67 ^eB^ ± 0.12	4.33 ^fC^ ± 0.06	5.23 ^fD^ ± 0.12	5.83 ^eE^ ± 0.06
**CE + HEO 0.5%**	2.37 ^cA^ ± 0.12	3.00 ^gB^ ± 0.10	3.87 ^gC^ ± 0.21	5.00 ^gD^ ± 0.10	5.90 ^fE^ ± 0.17	3.27 ^aA^ ± 0.06	3.73 ^fB^ ± 0.23	4.00 ^gC^ ± 0.17	4.60 ^gD^ ± 0.10	5.63 ^fE^ ± 0.06
**CE + HEO 1%**	2.47 ^bA^ ± 0.06	2.93 ^fB^ ± 0.12	3.60 ^hC^ ± 0.10	4.30 ^hD^ ± 0.17	5.70 ^gE^ ± 0.17	3.07 ^eA^ ± 0.12	3.40 ^gB^ ± 0.10	3.93 ^hC^ ± 0.12	4.30 ^hD^ ± 0.17	5.40 ^gE^ ± 0.17
**NE + HEO 0.5%**	2.43 ^bA^ ± 0.15	2.87 ^gB^ ± 0.06	3.33 ^lC^ ± 0.21	3.97 ^lD^ ± 0.06	4.87 ^hE^ ± 0.26	3.13 ^fA^ ± 0.12	3.40 ^gB^ ± 0.17	3.80 ^lC^ ± 0.10	4.00 ^lD^ ± 0.0	4.87 ^hE^ ± 0.06
**NE + HEO 1%**	2.37 ^cA^ ± 0.12	2.50 ^hB^ ± 0.10	3.07 ^mC^ ± 0.06	3.40 ^mD^ ± 0.17	4.40 ^lE^ ± 0.36	3.17 ^gA^ ± 0.06	3.30 ^hB^ ± 0.0	3.63 ^mC^ ± 0.06	3.77 ^mD^ ± 0.06	4.03 ^lE^ ± 0.06

The same uppercase letter within a column or the same lower-case letter within a row are not statistically different (p < 0.05).

### 3.5 Sensory evaluation

During the evaluation of sensory average (odor, color, texture and overall acceptance) it was found that the storage time for all samples had a significant effect (p < 0.05) on color, texture and overall acceptance (Tables 7). It has been found that in previous studies, the sensory profile of shrimp is considered acceptable if the sensory score is higher than 5.0 (Homayonpour et al., 2021, Homayounpour et al., 2020, Meilgaard et al., 1991, Pabast et al., 2018). The sensory evaluation score of shrimp decreased in all treatment groups during storage, and the sensory scores of the control samples decreased more than others (p < 0.05). At the end of the study period (12 days), the lowest sensory evaluation scores were recorded for the control samples (odor, texture, color and overall), and the highest sensory evaluation scores were reported for the NE-HEO 1% samples (odor, texture, color and overall). The sensory evaluation score of nano form samples was the best. A significant difference between the observations could be explained by a protective coating on shrimp by chitosan and HEO, which effectively prevented microbial growth in shrimp and reduced the protein degradation rate as well as the accumulation of volatile substances in shrimp (Aşik and Candoğan, 2014, Mohan et al., 2012). The Mohan et al. study showed that treatment with chitosan (1 and 2%) had a positive effect on extending the shelf life of Indian oil sardine (Mohan et al., 2012). A shelf life of one week analogous to the present observation for untreated sardine was stated for Indian oil sardine, Indian mackerel (Surendran et al., 1989), and Mediterranean anchovies (Pons-Sánchez-Cascado, Vidal-Carou, Nunes, & Veciana-Nogues, 2006), whereas relatively higher shelf life was reported for Atlantic sardine (Ababouch et al., 1996), pearl spot and black pomfret (Manju, Gopal, Jose, Ravishankar, & Kumar, 2007), Jack mackerel (Ryder, Buisson, Scott, & Fletcher, 1984), tilapia (Reddy, Schreiber, Buzard, Skinner, & Armstrong, 1994), seer fish (Mohan, Ravishankar, Gopal, Lalitha, & Kumar, 2010), eel (Özogul, Özogul, & Gökbulut, 2006), catfish (Mohan, Ravishankar, & Srinivasagopal, 2008) and sardine fish (Homayounpour et al., 2020).

**Table 7:** Predominant scores of sensory (1 to 9) for odour, texture, color and overall of shrimp samples throughout 12 days storing at 4 °C (mean). The higher the score, the quality is better and vice versa.

**Day/Treatment**	**Odor**					**Texture**					**Color**					**Overall**				
	0	2	4	8	12	0	2	4	8	12	0	2	4	8	12	0	2	4	8	12
**CON**	9^aA^	6.1 ^aB^	4.1^aC^	1.4^aD^	1.0 ^aE^	9^aA^	7.4 ^aB^	7.0 ^aC^	6.4 ^aD^	3.9 ^aE^	9^aA^	6.8 ^aB^	5.4 ^aC^	3.5 ^aD^	2.6 ^aE^	9^aA^	6.95 ^aB^	5.01 ^aD^	3.33 ^aE^	2.38
**CH**	9 ^aA^	6.2 ^bB^	4.4 ^bC^	1.6 ^bD^	1.2 ^bE^	9 ^aA^	8.0 ^aB^	7.4 ^bC^	6.3 ^bD^	5.2 ^bE^	9 ^aA^	6.8 ^bB^	5.4 ^bC^	3.5 ^bD^	2.6 **^v^**^E^	9 ^aA^	7.33 ^bB^	5.12 ^bD^	3.67 ^bE^	3.29
**NCH**	9 ^aA^	6.3 ^cB^	3.3 ^cC^	1.7 ^cD^	1.4 ^cE^	9 ^aA^	6.9 ^cB^	6.5 ^cC^	5.9 ^cD^	5.5 ^cE^	9 ^aA^	7.4 ^cB^	6 ^cC^	5.4 ^cD^	3.6 ^cE^	9 ^aA^	7.38 ^cB^	5.67 ^cD^	4.81 ^cE^	4.52
**SMS**	9 ^aA^	6.2 ^bB^	4.6 ^dC^	3.5 ^dD^	1.8 ^dE^	9 ^aA^	7.6 ^dB^	7.3 ^dC^	6.6 ^dD^	5.9 ^dE^	9 ^aA^	9^dB^	8 ^dC^	6.9 ^dD^	5.9 ^dE^	9 ^aA^	8.00 ^dB^	5.62 ^dD^	4.62 ^dE^	4.29
**HEO 0.5%**	9 ^aA^	6.4 ^dB^	5.4 ^eC^	2.6 ^eD^	1.3 ^eE^	9 ^aA^	7.6 ^dB^	7.3 ^dC^	6.5 ^eD^	6.0 ^eE^	9 ^aA^	7.7 ^eB^	6.4 ^eC^	5.7 ^eD^	5 ^eE^	9 ^aA^	7.62 ^eB^	5.62 ^dD^	5.38 ^eE^	4.90
**HEO 1%**	9 ^aA^	7.0 ^eB^	6.3 ^fC^	3.3 ^fD^	1.7 ^fE^	9 ^aA^	8.0 ^eB^	7.4 ^bC^	6.7 ^fD^	6.3 ^fE^	9 ^aA^	8.4 ^fB^	6.6 ^eC^	6 ^fD^	5.4 ^fE^	9 ^aA^	8.00 ^fB^	6.63 ^eD^	5.57 ^fE^	5.29
**CE + HEO 0.5%**	9 ^aA^	6.8^fB^	6.3 ^fC^	4.4 ^gD^	2.7 ^gE^	9 ^aA^	7.7 ^fB^	7.3 ^eC^	6.5 ^eD^	6.4 ^gE^	9 ^aA^	8.4 ^fB^	8 ^aC^	5.6 ^gD^	5.4 ^fE^	9 ^aA^	7.62 ^gB^	6.05 ^fD^	5.29 ^gE^	4.90
**CE + HEO 1%**	9 ^aA^	6.8 ^fB^	6.1 ^gC^	4.4 ^gD^	3.0 ^hE^	9 ^aA^	7.7 ^fB^	7.3 ^fC^	6.3 ^bD^	5.7 ^hE^	9 ^aA^	8.3 ^gB^	7.9 ^dC^	6.6 ^hD^	6.1 ^gE^	9 ^aA^	7.67 ^hB^	6.67 ^gD^	5.62 ^hE^	5.38
**NE + HEO 0.5%**	9 ^aA^	9.0 ^gA^	7.4 ^hC^	4.6 ^hD^	3.7 ^lE^	9 ^aA^	8.3 ^gB^	7.6 ^gC^	7.3 ^fD^	6.7 ^lE^	9 ^aA^	8.7 ^hB^	7.6 ^fC^	7.4 ^lD^	6.5 ^hE^	9 ^aA^	8.33 ^lB^	7.33 ^hD^	6.86 ^lE^	5.95
**NE + HEO 1%**	9 ^aA^	9.0 ^gA^	7.5 ^lC^	5.3 ^lD^	4.4 ^mE^	9 ^aA^	8.7 ^hB^	7.7 ^hC^	7.3 ^fD^	6.7 ^lE^	9 ^aA^	9 ^lA^	8.6 ^lC^	7.7 ^mD^	7.4 ^lE^	9 ^aA^	8.33 ^lB^	7.33 ^hD^	6.90 ^mE^	6.67

The same uppercase letter within a column or the same lower-case letter within a row are not statistically different (p < 0.05).

## 4 Conclusion

This is the first study to examine the effects of *Hyssopus Officinalis* essential oil (in different treatments) on shrimp storage. CH, NCH, SMS, HEO 0.5 %, HEO 1 %, CE + HEO 0.5 %, CE + HEO 1%, NE + HEO 0.5 %, and NE + HEO 1 % were all effective in protecting shrimp during storage. In microbial, chemical, and sensory evaluations, chitosan nanoemulsion coatings containing EO had the greatest effect compared to other treatments, and NE + HEO 1 % was more effective than others. Sodium metabisulfite performed better than control, CH, and NCH samples, but was less effective than other treatments (HEO 0.5 %, HEO 1%, CE + HEO 0.5 %, CE + HEO 1%, NE + HEO 0.5 %, and NE + HEO 1%). Thus, according to the results of this study, essential oils such as *Hyssopus officinalis* essential oil, especially in the form of chitosan nanoemulsion coating containing EO, should be used to store seafood such as shrimp at 4 °C. One of the limitations of this research is the lack of examination of *Hyssopus officinalis* plant extract forms, water or ethanolic extracts, on shrimp preservation. And it can be suggested that in future studies, the effects of the extract of this plant (in aqueous and ethanolic form) should also be investigated.

## CRediT authorship contribution statement

**Abbas Mehraie:** Methodology, Formal analysis, Resources, Writing – original draft. **Saied Khanzadi:** Conceptualization, Supervision, Funding acquisition, Project administration, Writing – review & editing. **Mohammad Hashemi:** Visualization, Investigation, Methodology, Software, Validation. **Mohammad Azizzadeh:** Methodology, Software, Validation, Data curation, Writing – original draft.

## Declaration of Competing Interest

The authors declare that they have no known competing financial interests or personal relationships that could have appeared to influence the work reported in this paper.

## Data Availability

Data will be made available on request.
